# Spectral imaging enables contrast agent–free real-time ischemia monitoring in laparoscopic surgery

**DOI:** 10.1126/sciadv.add6778

**Published:** 2023-03-10

**Authors:** Leonardo Ayala, Tim J. Adler, Silvia Seidlitz, Sebastian Wirkert, Christina Engels, Alexander Seitel, Jan Sellner, Alexey Aksenov, Matthias Bodenbach, Pia Bader, Sebastian Baron, Anant Vemuri, Manuel Wiesenfarth, Nicholas Schreck, Diana Mindroc, Minu Tizabi, Sebastian Pirmann, Brittaney Everitt, Annette Kopp-Schneider, Dogu Teber, Lena Maier-Hein

**Affiliations:** ^1^ Division of Intelligent Medical Systems, German Cancer Research Center (DKFZ), Heidelberg, Germany.; ^2^ Medical Faculty, Heidelberg University, Heidelberg, Germany.; ^3^ Faculty of Mathematics and Computer Science, Heidelberg University, Heidelberg, Germany.; ^4^ Helmholtz Information and Data Science School for Health, Karlsruhe/Heidelberg, Germany.; ^5^ Städtisches Klinikum Karlsruhe, Karlsruhe, Germany.; ^6^ Division of Biostatistics, German Cancer Research Center (DKFZ), Heidelberg, Germany.

## Abstract

Laparoscopic surgery has evolved as a key technique for cancer diagnosis and therapy. While characterization of the tissue perfusion is crucial in various procedures, such as partial nephrectomy, doing so by means of visual inspection remains highly challenging. We developed a laparoscopic real-time multispectral imaging system featuring a compact and lightweight multispectral camera and the possibility to complement the conventional surgical view of the patient with functional information at a video rate of 25 Hz. To enable contrast agent–free ischemia monitoring during laparoscopic partial nephrectomy, we phrase the problem of ischemia detection as an out-of-distribution detection problem that does not rely on data from any other patient and uses an ensemble of invertible neural networks at its core. An in-human trial demonstrates the feasibility of our approach and highlights the potential of spectral imaging combined with advanced deep learning–based analysis tools for fast, efficient, reliable, and safe functional laparoscopic imaging.

## Teaser

Assessing organ blood supply in real time is crucial in minimally invasive surgery and spectral imaging now makes it possible.

## INTRODUCTION

Replacing traditional open surgery with minimally invasive techniques for complicated interventions such as tumor resection is one of the most important challenges in modern health care. Conventionally used RGB (red, green, and blue) camera-based laparoscopes, however, are ill-suited for these demands. Their mode of operation is based on mimicking the human eye by collecting light in the aforementioned three broad regions of the optical spectrum; as a consequence, precise tissue differentiation and assessment of organ function remain largely inaccessible. Yet, obtaining real-time functional tissue information is crucial for many key procedures in minimally invasive surgery.

**Fig. 1.**
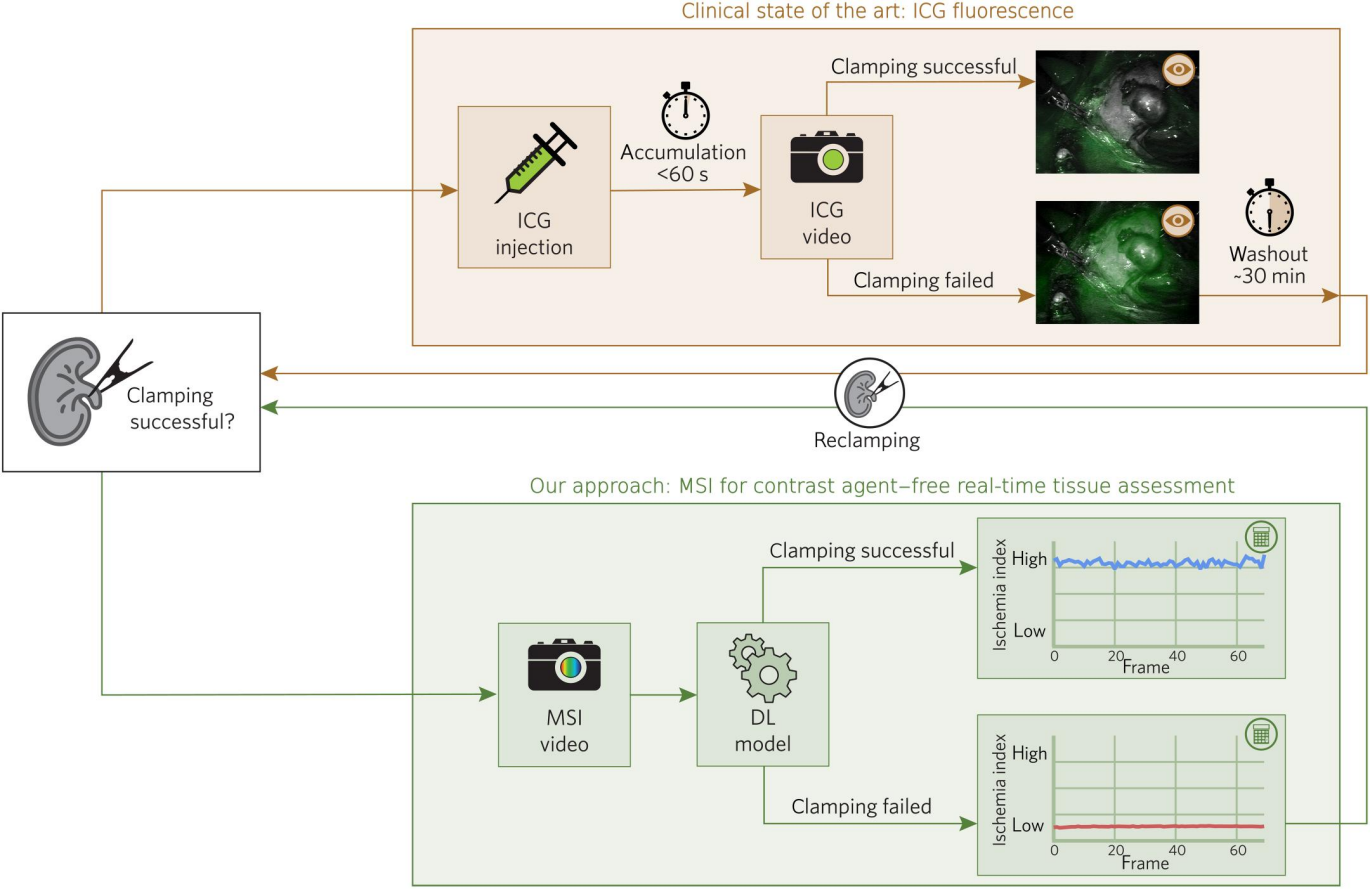
Our MSI-based approach enables continuous, contrast agent–free, real-time ischemia monitoring in laparoscopic surgery. The clinical state of the art (brown) to verify successful ischemia induction via clamping of arteries is based on ICG imaging: Ischemic tissue is characterized by a lack of fluorescent signal, whereas perfused tissue fluoresces. In the case of unsuccessful clamping, the test can only be repeated after a washout period of about 30 min. Our approach (green) is based on noninvasive and contrast agent–free MSI at video rate. DL models trained on MSI video sequences before clamping are capable of detecting ischemic tissue areas as outliers in real time, as detailed in (Fig. 3).

A common necessity of laparoscopic surgeries, for example, is stopping the blood flow to a specific organ or tissue region by clamping the arteries responsible for blood supply. This process, commonly referred to as ischemia induction, prevents excessive bleeding of patients (*1*) and is performed in various procedures, including partial nephrectomy, organ transplantation, and anastomosis. After clamping the main arteries, it is highly challenging to assess the perfusion state of the tissue solely based on the available RGB video stream. This especially holds true for selective clamping of a segmental artery, in which ischemia is induced only in the cancerous part of the kidney during partial nephrectomy (*2*, *3*). The most common approach to ensure correct clamping is based on indocyanine green (ICG) fluorescence (Fig. 1): After ICG is injected into the bloodstream, it binds to plasma proteins. The bound ICG travels through the bloodstream and accumulates in the internal organs, especially in the kidney and liver, within a minute (*4*, *5*). Lack of a fluorescent signal thus corresponds to lack of perfusion. However, because of long washout periods of about 30 min, this test is not easily repeatable if the wrong segment has been clamped (*5*) or if the clamping procedure was done improperly, as illustrated in Fig. 1. Furthermore, it requires a contrast agent to be injected into the bloodstream. Although ICG injection is generally regarded as a safe procedure, cases with severe complications such as anaphylactic shock have been observed (*6*).

Spectral imaging is a promising alternative approach to improving surgical vision (*7*). This technique removes the arbitrary restriction of recording only three broad spectral bands (RGB) by capturing an n-dimensional feature vector for each camera pixel, where each dimension corresponds to a comparatively narrower spectral band. As different tissue structures have unique optical scattering and absorption properties, knowledge of these optical properties along with spectral measurement data can potentially provide important information on tissue morphology (*8*–*10*) and function (*11*, *12*). The term multispectral imaging (MSI) is used when only a few bands (up to dozens) are recorded, while hyperspectral imaging (HSI) refers to hundreds of bands being recorded (*7*).

Despite the general success of MSI and HSI (*7*, *13*–*24*), applications in the operating room (OR) have been limited. Some of the main reasons why spectral imaging has not yet found its way into surgical practice are related to image acquisition time, processing time, and size of the available devices (*7*). Many available MSI/HSI camera systems are large (14 to 50 cm) and/or take several seconds (2 to 8 s) to record and process one image (*24*–*28*). To the best of our knowledge, the only laparoscopic spectral device proposed for clinical use so far (*26*) takes around 5 s to record one hyperspectral image, which prevents real-time application. In consequence, clinical success stories in spectral imaging for minimally invasive surgery are still lacking. Specifically, we are not aware of any clinical study in the broader context of real-time perfusion monitoring based on spectral imaging in laparoscopic surgery.

**Fig. 2.**
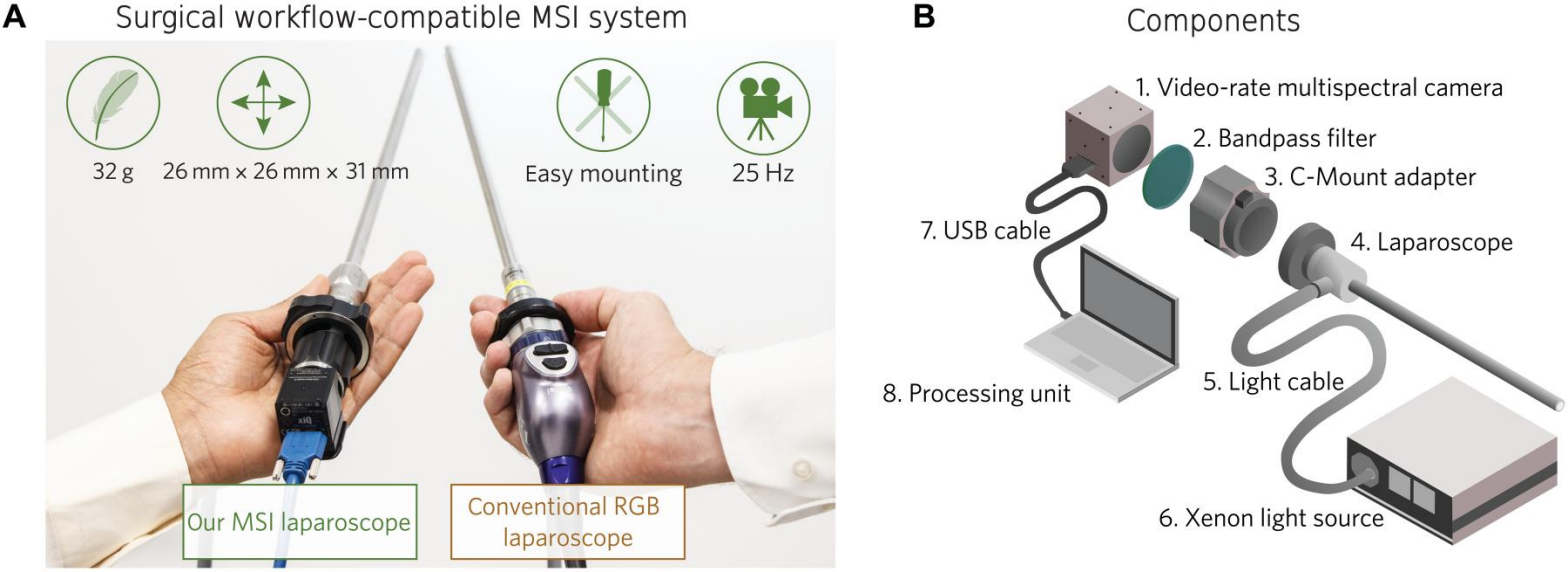
Our MSI system for ischemia monitoring is comparable to current standard equipment in terms of size and weight. (**A**) Proposed spectral imaging–based laparoscope (left) compared to standard RGB laparoscope (right). (**B**) Schematic representation of the system developed for spectral tissue analysis in laparoscopic surgery (see the “Multispectral imaging system” section). The dimensions of the laptop and the light source have been scaled down for visualization purposes. USB, universal serial bus.

**Fig. 3.**
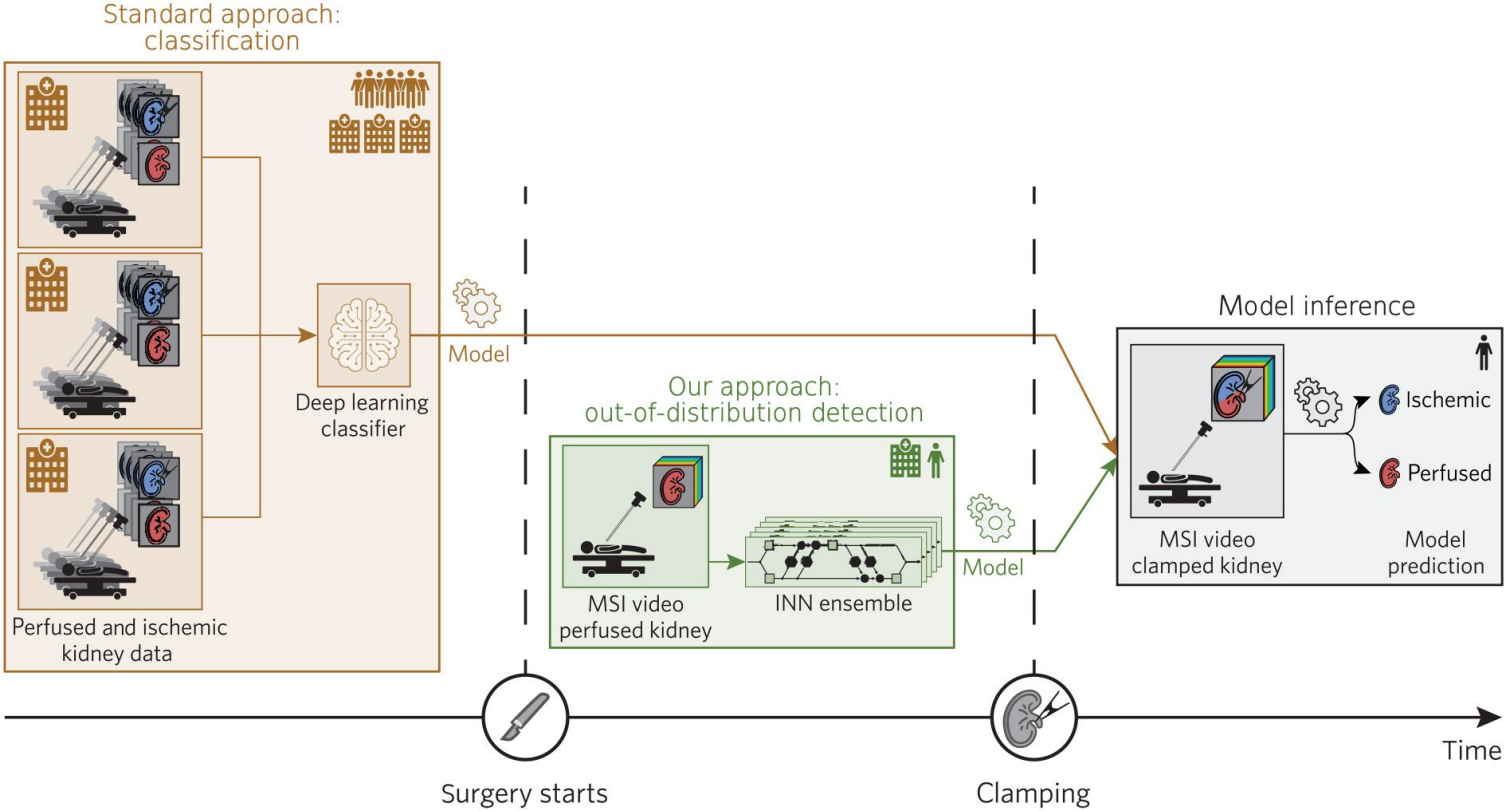
We treat ischemia monitoring based on MSI as an OoD task, which only needs training data from one single patient. Traditional DL methods (brown) require large amounts of patient data to train a model, while our method (green) only needs data from a single patient. Using an ensemble of INNs as a core component, our algorithm computes the likelihood of ischemia based on a short (several seconds) video sequence acquired at the beginning of each surgery. An important feature of our approach is that the entire training and inference process can be performed during a surgical procedure.

We address this gap in the literature with the following contributions:

1) Video-rate MSI system (Fig. 2): We present the real-time (25 Hz) laparoscopic MSI system applied in patient studies. It features a compact (26 mm by 26 mm by 31 mm) and lightweight (32 g) MSI camera that can be connected to standard laparoscopes via a widely used C-Mount adapter and operates with clinical light sources.

2) Deep learning (DL)–based algorithm for real-time ischemia detection (see Fig. 3): To overcome the need of large amounts of training data required by traditional discriminative machine learning methods, we phrase the problem of ischemia detection as an out-of-distribution (OoD) detection problem that does not rely on data from any other patient (patent pending). Using an ensemble of invertible neural networks (INNs) as a core component, our algorithm is trained to compute the likelihood of ischemia based on a short (several seconds) video sequence acquired at the beginning of each surgery.

3) In-human study: We present an in-human study demonstrating that monitoring kidney ischemia in real time is now possible.

## RESULTS

### In-human application of video-rate multispectral laparoscope

The proposed system enables us to pioneer clinical video-rate MSI in laparoscopy. As shown in Fig. 2, it comprises (i) a snapshot MSI camera, (ii) a 335- to 610-nm bandpass filter, (iii) a C-Mount adapter with adjustable focal length, (iv) a standard surgical laparoscope, (v) a surgical light cable, (vi) a xenon light source, and (vii) a universal serial bus cable to connect the MSI camera to the (viii) processing unit (see the “Multispectral imaging system” section). By design, the camera covers the wavelength range in which oxyhemoglobin (HbO_2_) and deoxyhemoglobin (Hb) are the main absorbers of light in internal organs. As oxygenation and blood volume fraction are defined in terms of HbO2 and Hb, this wavelength range is particularly well-suited for deriving estimates for these two tissue parameters. As ischemia is characterized by a reduction of tissue oxygenation and blood volume fraction (*29*), our hypothesis was therefore that it would be well-suited for perfusion monitoring in general and ischemia detection in particular. Because of the compact (26 mm by 26 mm by 31 mm) design, the camera does not add hardware complexity to the OR (see Fig. 2A). Furthermore, the camera is extremely lightweight (32 g), thus enabling easy handling of the device over long periods of time. The system operates at a frame rate of 25 Hz. To compensate for tissue and camera motion during image acquisition, we developed a DL-based algorithm capable of tracking regions of interest (ROIs) within an MSI video (see the “Implementation details” section). The algorithm was successfully applied in 10 patients undergoing partial nephrectomy and served as a prerequisite for further analysis and for computing the DL-based ischemia index. The results of our analysis are exemplary illustrated in this video.

### High interpatient variability for kidney tissue

**Fig. 4.**
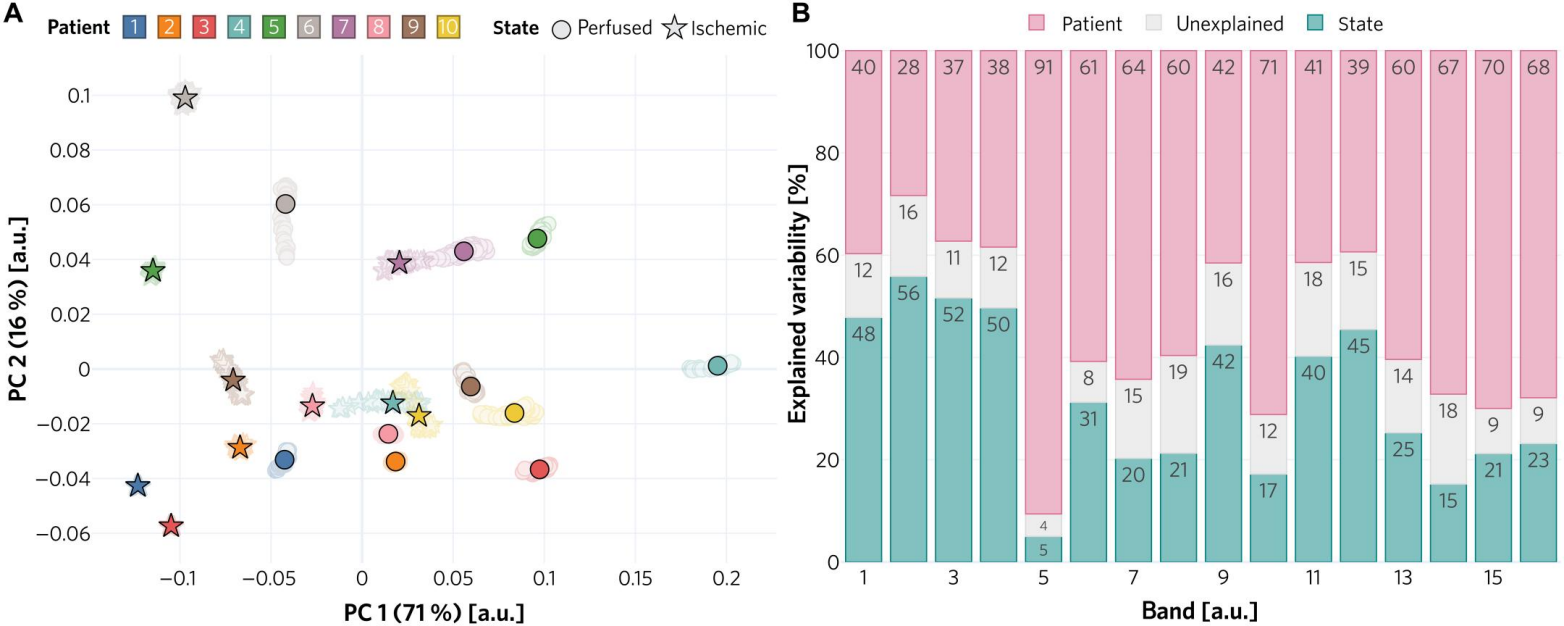
High tissue heterogeneity across subjects motivated our personalized approach. Both the (A) PCA and (B) the mixed-model analysis demonstrate the high interpatient variability of spectral tissue measurements. (**A**) The fact that different tissue states cluster within a subject but do not form a uniform cluster across subjects motivated us to phrase the challenge of ischemia detection as an OoD problem. Solid markers show the cluster centers, transparent markers show the raw data points, and the axis labels denote the explained variance of the corresponding PC. (**B**) Explained variance for the 16 bands of the MSI camera, depicted in Fig. 6.

High interpatient variability generally suggests poor generalizability of supervised learning algorithms. In a recent porcine study, we showed that the greatest source of variability related to spectral images of organs acquired from healthy animals is the organ under observation rather than the recorded individual or specific acquisition conditions (*30*). This enabled us to develop a highly accurate supervised DL algorithm for fully automatic organ classification (*31*). However, an analogous analysis applied to the data of the clinical study presented here revealed that kidney tissue of patients undergoing partial nephrectomy is highly heterogeneous. As illustrated in Fig. 4, when reducing the full spectral information to two dimensions via principal components analysis (PCA) (*32*), the measurements from different patients gather within clear visual clusters for each state [indicated by a circle (perfused) or star (ischemic) in Fig. 4A], while a clustering of different tissue states across patients cannot be observed (circles and stars do not form clear clusters). Note in this context that the first two principal components (PCs) capture 87% of the variation. Furthermore, according to a mixed-model analysis, most variability in the measurements can be explained by the individual patient rather than—as would be desired—the tissue state (see Fig. 4B). In consequence, tackling the challenge of ischemia detection with a traditional supervised algorithm trained on a small dataset would come with a high risk of poor generalization capabilities to unseen individuals. In addition, (slight) changes in the acquisition setup might require complete retraining of the method on the entire patient database. This motivated our personalized approach to ischemia detection, illustrated in Fig. 3.

### Novel DL-based ischemia index captures ischemia state

**Fig. 5.**
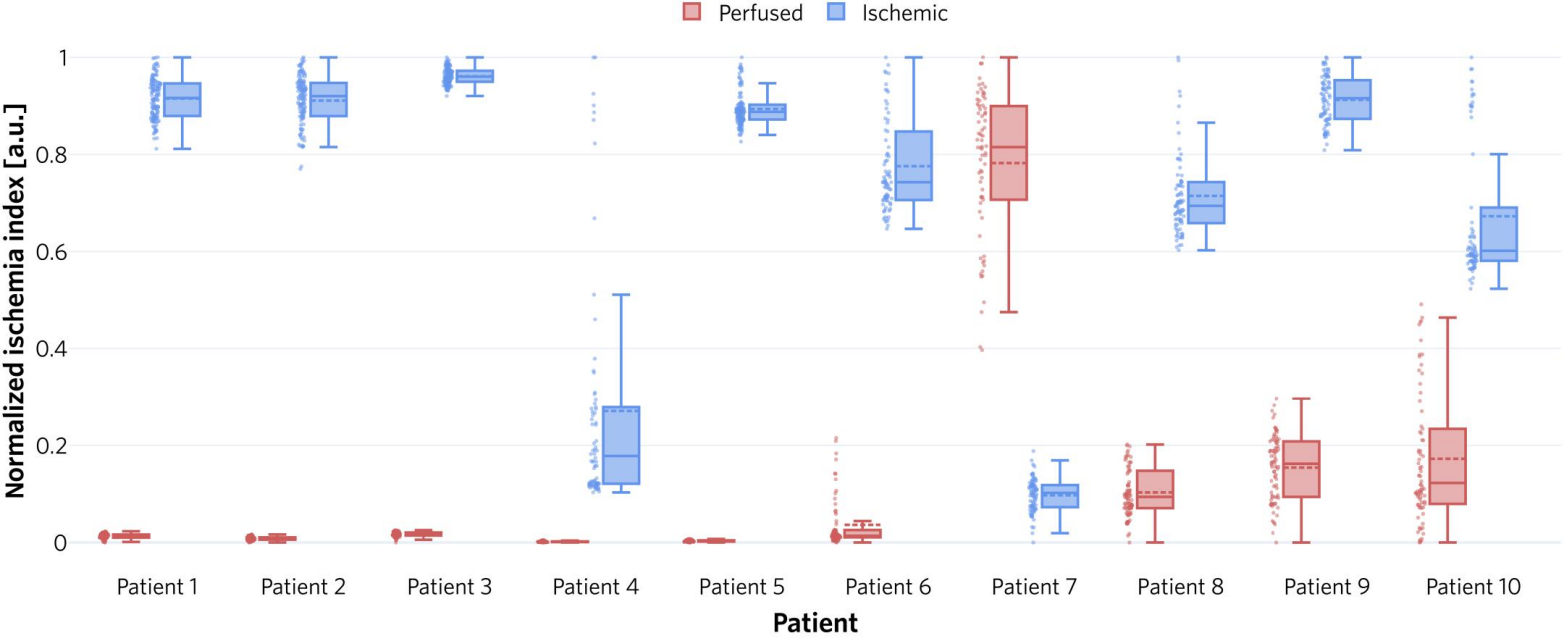
Our approach is capable of discriminating between ischemic and perfused kidney states. We calculated the ischemia index for every frame in video sequences of perfused and ischemic kidney separately for each patient (see the “Algorithm for live ischemia monitoring” section) and generated corresponding dot and box plots. The boxes show the interquartile range with the median (solid line) and mean (dashed line), while the whiskers extend up to 1.5 the interquartile range. Min-max normalization was performed for clarity of presentation.

To overcome the limitations of supervised approaches in the presence of high tissue heterogeneity, we developed a personalized approach to ischemia detection that solely requires data of the patient undergoing surgery (see Fig. 3). Specifically, we phrase the problem of ischemia detection as an OoD detection problem and assess the perfusion state with a custom-designed ischemia index that relies on an ensemble of INNs as the core component. As detailed in the “Algorithm for live ischemia monitoring” section, the ensemble is trained to estimate the density of the distribution of perfused spectra from a short (several seconds) video sequence acquired at the beginning of each surgery. At inference time, the density is evaluated on new spectra and the ensemble predictions are aggregated using the widely applicable information criterion (WAIC) (*33*). In short, the WAIC bases the OoD value for a given (new) spectrum on two potentially complementary indicators: the density of the spectrum (given by the mean log likelihood predicted by the ensemble) and the uncertainty of the ensemble predictions at the location of the new spectrum (given by the variance). If either the density is low or the uncertainty is high, WAIC is high, which indicates OoD data. The ischemia index, computed for each image, represents the spatially aggregated WAIC values. According to our patient study with 10 patients undergoing partial nephrectomy, in which ICG fluorescence served as the gold standard method, our novel ischemia index classifies the perfusion state with high accuracy (see Fig. 5). In almost all patients, the data corresponding to ischemic tissue could be perfectly separated from those corresponding to perfused tissue. This led to a median/mean area under the receiver operating curve (AU-ROC) of 1.0/0.9 obtained for the *n* = 10 patients. With conventional RGB camera data, such a clear separation would not be possible, as confirmed by our data. When applying our method to reconstructed RGB data, the ischemia indices corresponding to different time points showed substantial overlap for the vast majority of patients (8 of 10), as illustrated in fig. S2. While the patient-individual training took 30 s per network on average, the actual index calculation runs at 140 Hz per ROI on the kidney and thus in real time.

## DISCUSSION

MSI is on the verge of opening up entirely new avenues in the early detection, diagnosis, and monitoring of diseases ranging from cancer to cardiovascular and inflammatory diseases, particularly when applied in modern interventional health care such as minimally invasive surgery. To the best of our knowledge, this work presents the real-time application and analysis of MSI in laparoscopic surgery on human subjects and in a clinical setting. On the basis of a newly developed video-rate multispectral laparoscopic imaging system, our approach presents three key advantages over the current state of the art in interventional ischemia detection:

1) No contrast agent: Our method is completely noninvasive and does not require the administration of ICG or any other contrast agent. This reduces intervention time and costs and enhances patient safety by reducing complication risks (e.g., anaphylactic shock).

2) Multiple uses per surgery possible: In case of a failed ischemia induction attempt, our method can be reapplied instantly. There is no washout period.

3) Video-rate ischemia monitoring: The proposed ischemia index can be computed in real time, making continuous ischemia monitoring feasible, whereas the classical approach allows for only a single measurement per surgery.

In the following sections, we discuss our hardware choices to achieve video-rate ischemia monitoring, the consequences of high tissue heterogeneity, our approach to ischemia detection, and a comparison between our proposed approach and current clinical practice.

### MSI system design

We developed a real-time MSI system for laparoscopic surgery that was applied in a patient study. The key strengths of our imaging system are the high acquisition speed (above 25 MSI images per second), the low weight (32 g), the small size (camera cube with an edge size of 26 mm by 26 mm by 31 mm), and the fact that it operates with a standard clinical light source, all of which allow for smooth integration into the surgical workflow. Furthermore, all relevant changes made compared to the standard clinical setup concern components that are attached to the laparoscope and, as such, never touch the patient, which minimizes the patient risk introduced by a new imaging device. A key design choice was the integration of a bandpass filter to counteract oversaturation resulting from strong signals in the red region of the visible spectrum.

Previous work on clinical spectral imaging has focused on systems for open surgery with acquisition times of several seconds (*24*–*28*). Preclinical multispectral or hyperspectral systems proposed in the specific context of laparoscopy have typically been based on sequential image acquisition (*11*, *28*, *34*, *35*), e.g., using the filter wheel technology (*11*, *20*, *34*), and thus come with the risk of motion artifacts and the lack of video-rate acquisition capabilities. The snapshot technique applied here has, in parallel, been explored by other authors (*36*) in the context of open surgery. Overall, we are not aware of any real-time MSI or HSI laparoscopic system that has been applied in humans so far.

**Fig. 6.**
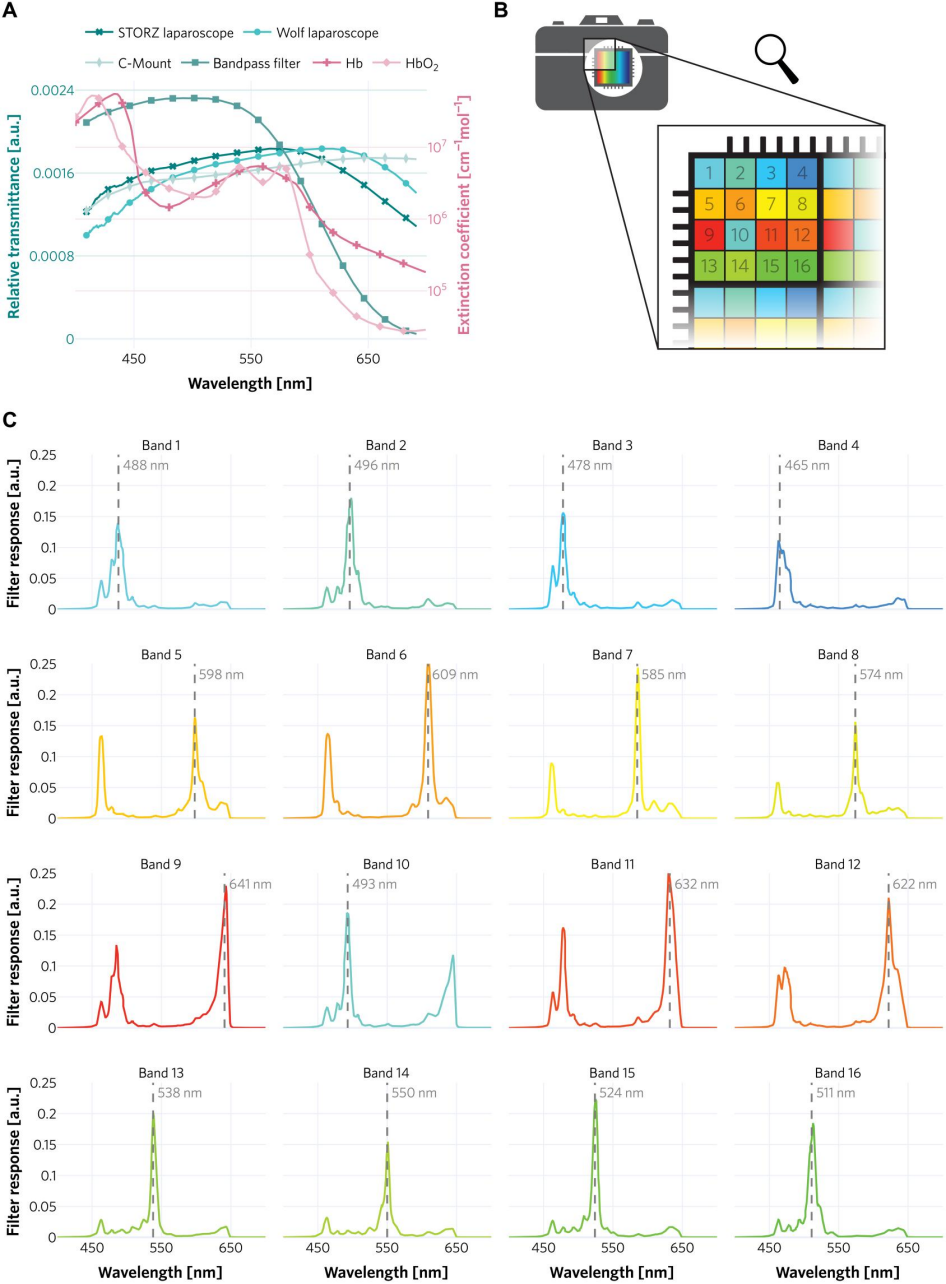
Optical properties of our multispectral system. (**A**) ℓ_1_-Normalized transmission spectra of the laparoscopes, bandpass filter, and C-Mount adapter are shown in the left axis. Extinction coefficients of HbO_2_ and Hb are shown in the right axis. The bandpass filter mainly filters light in the low-wavelength region where blood absorbs the most (below ≈600 nm), thus making the spectral power distribution that reaches the camera detector more uniform across wavelengths. (**B**) Representation of the 4 × 4 mosaic pattern of the multispectral camera sensor. Each colored square represents a different filter; these filters form a 4 × 4 pattern that extends over the whole image. (**C**) Filter responses of the multispectral camera bands. Some bands, such as 5 to 12, show two peaks in the spectral response, which are referred to as “second-order” peaks.

While the snapshot technique is the key enabler for fast image recordings, it also comes with several limitations. The second-order peaks of the MSI camera filters shown in Fig. 6 can potentially present challenges to image analysis. Given the fact that these second-order peaks are located in those regions in which HbO_2_ and Hb are the primary absorbers and can potentially help derive relevant tissue properties, they need to be taken into account when the target application is quantitative perfusion or oxygenation estimation. For example, applying the Beer-Lambert law (*34*, *37*) to our data yielded implausible values (e.g., oxygenation above 100%), as discussed below. Our proposed method is not influenced by these second-order peaks because the INNs learn the density of the MSI data, regardless of the influence such peaks have on the reflectance spectra. More specifically, given that all image sequences are equally influenced by these second-order peaks, INNs encode such influences in the model and learn to predict our ischemia index based only on physiological changes and not on hardware configurations. It is worth mentioning that while the sensor of the multispectral camera that we use tiled multiple bands spatially, the sensor featured a relatively large pixel size of 5.5 μm that enabled us to record data with a high signal-to-noise ratio (SNR). More in detail, our measurements featured a median/mean SNR of 72/81 as illustrated in fig. S4.

For the present study, we chose a camera that operates in the visible spectrum. We made this choice mainly for regulatory reasons as it allowed us to use a standard clinically certified light source. However, the tissue spectrum in the infrared (IR) range is often very expressive (*10*), promising even better performance in estimating tissue parameters. It could thus be viable to explore this regime with a combination of cameras receptive in the IR range and IR optimized light sources. Another necessary adaptation of the camera system would be the capability to provide both a regular RGB image for human-interpretable visualization to the surgical staff and MSI data for the ischemia index computation. Note in this context that the spatial resolution of our current camera is already high, as demonstrated by the RGB images reconstructed from the MSI data (see fig. S3).

### Tissue heterogeneity and personalized approach

This work constitutes systematic in vivo spectral analysis of diseased kidney tissue. Our analysis clearly exposed the high interpatient variability of optical properties. A key finding was that the main contributor to the variance of spectral measurements is the patient rather than the perfusion state of the kidney. These results dim the hopes of successfully applying supervised machine learning algorithms as ischemia detectors under conditions of limited patient data.

Our findings regarding the heterogeneity are in stark contrast to recent findings in porcine organs (*30*), where the influence of the different specimen is small compared to other explanatory variables. A key difference between this study and the present work (apart from the species) is that for the porcine study all organs were healthy, whereas all of our human subjects underwent partial nephrectomy due to kidney cancer. This might be one factor explaining the high interpatient variability.

The limited number of patients included in our study makes it hard to determine the mechanism for the change in spectra. In particular, the combination of cancer type and comorbidities is unique for each patient. Furthermore, the complex environment of the OR offers a significant number of external factors that cannot be controlled. For example, the pose of the laparoscope relative to the kidney changes between each patient due to variables such as port placement, respiratory movements, different anatomy, etc. Because the laparoscope is operated freehand, additional motion from the surgeon is involved. Data normalization can only compensate for some of these factors. Overall, the described mismatch between sample size and the number of variable factors makes attribution of spectral variability to these factors hard. However, they are captured as the remaining unexplained variance in Fig. 4B. While a larger patient number would be desirable from a purely scientific standpoint, we needed to weigh the benefit against ethical considerations using uncertified equipment in the OR as well as further monetary and regulatory hurdles.

We observed that the explained variability of bands 5 and 10 that can be accounted for by changes in tissue state (perfused versus ischemic) is especially low in comparison to the variability explained by different patients (see Fig. 4). We attribute this to two main reasons: (i) The maxima of the camera filter responses corresponding to these bands are located at wavelengths where the extinction coefficient of Hb and HbO_2_ are very similar (see Fig. 6). This, in turn, leads to lower variability between tissue states. (ii) Band 5 is also affected by a second-order peak with a maximum filter response located at a wavelength where the extinction coefficients of Hb and HbO_2_ are considerably smaller, thus leading to higher reflectances and a lower influence of different tissue states (perfused versus ischemic) in the reflectance signal.

In the future, the incorporation of more explanatory variables (such as comorbidities) in our linear mixed model would allow for an in-depth understanding of how different factors affect spectral measurements. However, with the frequency of partial nephrectomies being limited, particularly in pandemic times, additional data acquisition is a slow process. Obtaining an MSI imaging device certified for clinical use can be considered a pivotal step in accelerating data acquisition for large-scale studies.

### Ischemia index

We introduced an entirely novel approach to ischemia detection that relies neither on potentially oversimplified modeling assumptions (such as the Beer-Lambert law) nor on a large patient cohort for algorithm training. Our results show that we can overcome difficulties posed by the high interpatient variability through rephrasing the problem of ischemia detection as an OoD detection problem. In the vein of personalized medicine, this process is solely dependent on data from the patient in question, which avoids the necessity for consideration of confounders introduced by different patients (*25*, *38*). This constitutes an extremely relevant feature in the face of increasing evidence that many research studies overestimate the performance of DL algorithms because of poor selection of test data relative to the training data (*39*, *40*). Furthermore, algorithms trained on data from a specific camera may not necessarily generalize to slightly adapted conditions (*25*, *41*). Although our model has to be retrained for every patient, application in the OR is feasible as only spectra of perfused tissue are required for training, and the training time is approximately 30 s per network (five networks). Such a training procedure can easily be performed at the beginning of each surgery before identification of the renal artery, thus avoiding delays in the surgical procedure. Furthermore, at a spectrum evaluation rate of 130 kHz, which translates to an individual ROI evaluation rate of 140 Hz, the inference is real-time capable.

The clinical state-of-the-art method for assessing ischemia in partial nephrectomy is based on ICG fluorescence. As summarized in Table 1, key advantages of our method are its noninvasiveness and real-time capability. A limitation of our approach can be seen in the fact that our algorithm requires a clean kidney to perform reliably. Excessive bleeding, scarring, or remaining fatty tissue on the kidney surface in particular may hinder the application of our method. On the other hand, this disadvantage may potentially be overcome by extending the wavelength range to the IR range, which is associated with deeper tissue penetration.

**Table 1.** Key characteristics of traditional ischemia monitoring with ICG injection and our proposed noninvasive method.

Feature	ICG	Our method
Standard light source	✘	✓
Standard camera	✘	✘
Low per use cost	✘	✓
Low investment cost	✘	✘
Contrast agent–free	✘	✓
Multiple use per surgery	✘	✓
Robust to tissue surface imperfections	✓	✘
Real time	✘	✓

The state-of-the-art approach to assessing perfusion based on spectral measurements in preclinical studies is to apply the Beer-Lambert law (see the “Implementation details” section) (*34*, *37*). However, this yielded implausible values (e.g., oxygenation above 100%). In addition, a clear separation of perfusion states could only be achieved in 50%/30% of the patients, depending on whether oxygenation or blood volume fraction estimation was used as a decision score.

Other model-based approaches to estimating physiological parameters, such as the one applied by the TIVITA cameras (Diaspective Vision GmbH, Am Salzhaff-Pepelow, Germany), rely on first- and second-order derivatives of the spectra (*35*). Such an approach thus requires fine-grained spectra and is potentially highly sensitive to noise. In consequence, its application has so far been restricted to HSI camera setups associated with long acquisition times.

In our own previous work, we presented a machine learning–based approach to physiological parameter quantification based on MSI data (*11*, *12*). To address the absence of a reliable reference method for generating labeled training data, we based our approach on simulations generated with Monte Carlo (MC) methods. To this end, we leveraged prior knowledge on tissue composition, optical properties, and light-tissue interaction to generate a large pool of synthetic spectra, labeled with corresponding ground truth physiological parameters. These data were then used to train a machine learning–based regressor (*11*, *12*, *42*), possibly after a domain adaptation step (*12*). We did not apply the method in this study as our kidney measurements were OoD compared to the simulations, which indicates that more work is needed for fully realistic spectra generation in the presence of pathologies. To overcome the lack of accurate prior knowledge as required by model-based approaches or the simulation-based model, we decided to explore the personalized method presented in this manuscript.

**Fig. 7.**
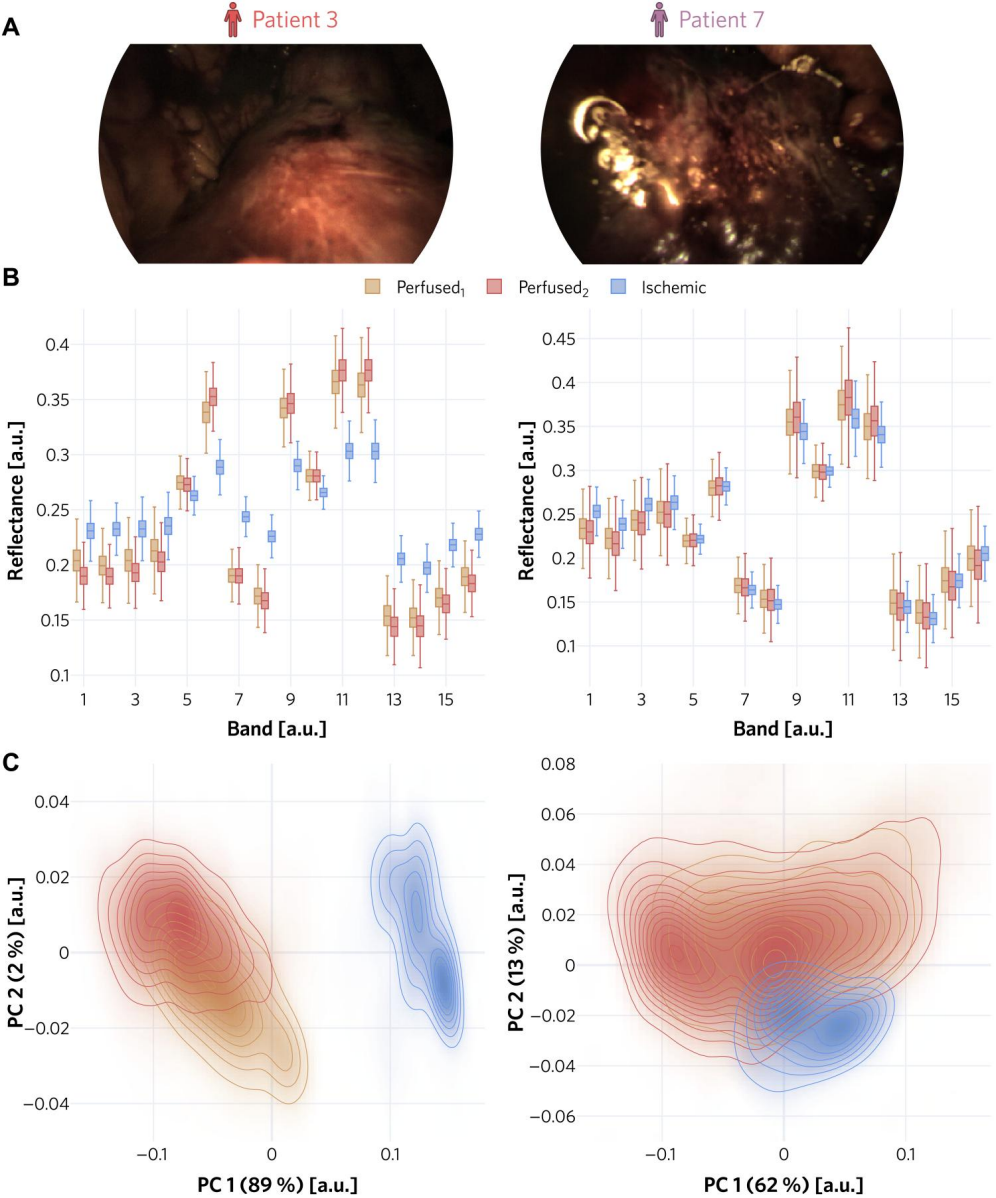
Comparison of a representative patient with patient 7 with respect to the algorithm input. (**A**) Example RGB images were selected from the perfused_2_ sequence to illustrate the unusual images acquired of the kidney of patient 7. (**B**) The reflectances of the representative patient (patient 3) differ clearly between perfused and ischemic tissue for the vast majority of camera bands, while no clear separation can be observed in patient 7. The boxes show the interquartile range with the median (solid line), while the whiskers extend up to 1.5 the interquartile range. (**C**) A KDE performed on the data resulting from a patient-specific PCA provides further explanation for why our method falsely detected the ischemic kidney data of patient 7 as inlier. The axis labels denote the explained variance of the corresponding PC.

While our method worked perfectly on 9 of 10 patients, it failed on patient 7. Further analysis revealed that the spectra between different kidney states did not substantially differ for this particular patient, as shown in Fig. 7. A kernel density estimation (KDE) performed on the data resulting from a patient-specific PCA showed a considerable overlap between tissue states. Furthermore, the perfused spectra exhibited higher variability than the ischemic spectra consistently across all bands. These two factors might explain why ischemic spectra were erroneously detected as in domain, with the support of the distribution of perfused spectra containing the support of the distribution of ischemic spectra. A possible reason for the similar spectra in perfused and ischemic state is the appearance of burned fatty tissue in the surface of the kidney, which can occur while removing fatty tissue with the da Vinci monopolar scissors and negatively affect tissue penetration. To mitigate problems related to tissue penetration and thus preclude failure of our method, we envision increasing the wavelength range of the camera and the light source in the future, as mentioned above. Because this change requires a new hardware setup (higher power light source) with regulatory approval, it remained beyond the scope of our current study. It should also be mentioned that the laparoscopic videos of patient 7 looked unusual from a clinical perspective (see Fig. 7A). As patient 7 also happened to be the only smoker among the participants of our study, we cannot rule out a possible effect on the measured spectra. Investigating the impact of comorbidities on MSI-based ischemia detection and potential failure cases was not within the scope of the present work but could be the subject of a more comprehensive study in the future.

Notably, patient 4 presented a particular case in our analysis. Because of data corruption of the white Spectralon reference measurements (see the “Automatic ischemia detection” section), we used the white measurements recorded during another surgery with an identical hardware setup (patient 3). Given that hardware, software, and Spectralon target were all identical, we did not expect a major influence of this procedure in the analysis of the data from patient 4. Experiments where the data from patient 4 were normalized using the white and dark references from other patients revealed that the variation across different normalization strategies is not substantial compared to the variance across kidney states (perfused and ischemic).

It is highly important to note that we achieved encouraging results although our algorithm was confronted with overly complex conditions. A specific challenge within this study was the fact that we needed to remove our laparoscope between training and testing data acquisitions. The reason was that we did not want to alter the clinical procedure more than necessary and thus based the clinical decision-making exclusively on a standard, certified (additional) RGB camera. In order not to require an additional port for our own laparoscope (which would have come at the cost of increased invasiveness), we used the port that is required for the clamping for our device. This made temporary retraction necessary, thus complicating the ischemia detection because analysis on the same ROI could not be guaranteed. Note that in some of the patients, it was not even manually possible to robustly locate the same tissue region before and after clamping because of the lack of landmarks and the altered pose of the laparoscope relative to the tissue. To ensure that our OoD detector does not merely detect different tissue regions (rather than tissue states), we took the design decision to base the analysis on two potentially disjoint ROIs. Furthermore, we designed the validation study in a way that we acquired data from perfused and ischemic tissue in a comparable manner, always after removal and reinsertion of the laparoscope. If the reason for OoD detection in the ischemic tissue was the change of tissue region, the algorithm would have failed on the testing sequences corresponding to perfused tissue (perfused_2_). In consequence, however, the task for the algorithm was overly complex. Rather than having to determine whether a perfusion change on a specific tissue region had occurred (by comparing identical tissue regions before and after the clamping attempt), the algorithm needed to determine whether the perfusion state of a specific region after the clamping attempt was different to that of another (potentially different) region before the clamping attempt. Notably, the challenge of interrupted image acquisition may easily be overcome in the future with a clinically certified MSI laparoscope, enabling MSI acquisition and physician interpretation at the same time. In consequence, our performance estimation can be considered conservative, and our method may achieve better results with a clinical system that allows for simultaneous spectral and RGB imaging.

In conclusion, we presented an in-human study demonstrating that intraoperatively monitoring kidney perfusion in real time is possible by exploiting a combination of spectral imaging and advanced DL-based analysis algorithms. Our study can be seen as a foray into the application of MSI in a clinical interventional setting.

## MATERIALS AND METHODS

This section presents the methods corresponding to the three primary contributions of our paper, namely, the MSI system (see the “Multispectral imaging system” section), the algorithm for ischemia detection (see the “Algorithm for live ischemia monitoring” section), and our clinical study for validating our approach (see the “Patient study” section). Implementation details on some of the modules and methods have been moved to a separate section (see the “Implementation details” section).

### Multispectral imaging system

Our MSI system is illustrated in Fig. 2 and comprises the following main components.

#### Multispectral camera

The core component is an MSI camera (MQ022HG-IM-SM4x4-VIS, XIMEA GmbH, Münster, Germany), which is small (26 mm by 26 mm by 31 mm) and light (32 g). It is based on the imec (Leuven, Belgium) mosaic snapshot sensor, which acquires 16 spectral bands at a single snapshot, using a 4 × 4 repeating mosaic pattern of filters (Fig. 6B); the spectral response of each filter is shown in Fig. 6C. Several of the bands show two peaks in the spectral response. These are caused by the measurement principle of the Fabry-Pérot filters (*43*), which lead to the so-called “second-order” peaks. The intensity of such peaks depends on the height of the optical cavity of the filters, the refractive index of the sensor material, and the cosine of the light incidence angle (*44*). The spatial resolution of the sensor is 272 × 512 pixels.

#### Surgery-specific components

Because of intrinsic optical tissue properties, the reflectance of human tissue in the red region (above ≈620 nm) is higher than that in the blue region (below ≈490 nm). To ensure more balanced camera counts and thus similar noise levels across different camera filters, a 335- to 610-nm bandpass filter (FGB37, Thorlabs Inc., Newton, NJ, USA) was placed between the C-Mount adapter and the multispectral camera. The C-Mount adapter (20200043, KARL STORZ SE & Co. KG, Tuttlingen, Germany) features an adjustable focal length, with a maximum of 38 mm. To enable the recording of MSI images during minimally invasive surgery, the camera was connected to standard 30° laparoscopes (26003BA, KARL STORZ SE & Co. KG, Tuttlingen, Germany and Panoview, Richard Wolf, Knittlingen, Germany) via the aforementioned C-Mount adapter. The ℓ_1_-normalized transmission spectra of the bandpass filter, the laparoscope, and the C-Mount adapter are shown in Fig. 6. The extinction coefficients of HbO_2_ and Hb are also shown as a reference (*45*). As light source, we chose a xenon light source (IP20, Richard Wolf GmbH, Knittlingen, Germany) as it provides brighter and more uniformly distributed light across different wavelengths in comparison to a halogen light source.

#### Image recording and storing

For image acquisition and recording, a standard laptop (Msi GE75 Raider 85G, Intel i7, NVIDIA RTX 2080) was used. A custom C++ software (not publicly available) based on the XIMEA Application Program Interface (XiAPI, XIMEA, Münster, Germany) was implemented and used to record the MSI images.

### Algorithm for live ischemia monitoring

We phrase the challenge of live ischemia monitoring as an OoD detection problem that only relies on data of the current patient. This mitigates the challenge of acquiring large amounts of annotated training data and the limited robustness, which traditional machine and DL approaches suffer from due to high variability of human tissue, acquisition protocols, camera setups, etc. Specifically, our algorithm is trained to compute the likelihood of normal tissue perfusion based on a (several seconds) video sequence acquired at the beginning of each surgery. In the following paragraphs, we review the principle of WAIC (*33*), explain how to leverage INNs to compute it in real time, and present our novel OoD-based ischemia index.

#### Widely applicable information criterion

While we are not aware of any previous work in OoD detection in the field of optical imaging, the topic of OoD has gained increasing interest in the machine learning community. We build our method upon the work by Choi *et al.* (*46*) who proposed WAIC as a means to measure the closeness of a new sample to the training distribution. In Watanabe (*33*), WAIC was defined as

$$\mathrm{WAIC}(x)=\mathrm{Va}r_{\text{Θ}}[\log P(x|\text{Θ})]-E_{\text{Θ}}[\log P(x|\text{Θ})]$$ (1)

where Θ represents the parameters of the model family that parameterize *P* and the variance, and expectation is taken over Θ ∼ *P*(Θ ∣*X*^tr^) of the training distribution *X*^tr^. Then, WAIC(*x*) estimates the proximity of a sample *x* to *X*^tr^. Choi *et al.* (*46*) suggested to use WAIC as a means for OoD in the setting of neural networks. WAIC is made up of two terms. The second term is the expectation over the negative log likelihood of a sample. The negative log likelihood is regularly used as an OoD score, the idea behind it being that OoD samples should lie in low-density regions of the in-distribution. However, this assumption is violated in higher dimensions, where samples close to the maximum of the log likelihood are drawn infrequently, too, due to the low volume of that region. WAIC addresses this problem by modifying the expectation term with a variance term (the first term in Eq. 2). In densely populated regions of the in-domain dataset, the training signal will pull the log likelihood estimates of the ensemble together, but for sparsely populated regions, there is no such signal, and because of the random initialization of the members, there is no reason to expect similar predictions. In other words, the variance between the ensemble predictions should be high in sparsely populated regions. This way, WAIC is able to detect OoD samples even in high-density but low-population regions. A major challenge related to the implementation of WAIC is the efficient computation of log*P*(*x*∣Θ). We propose applying INNs to be able to compute log*P*(*x*∣Θ) and thus WAIC(*x*) in real time during a surgical procedure.

#### INNs for computing WAIC

Our approach to computing log*P*(*x*∣Θ) is based on two steps: (i) leveraging INNs for converting a measurement into a space in which we can analytically compute the log likelihood and then (ii) applying the change of variable formula to obtain log*P*(*x*∣Θ). More specifically, we use INNs (*47*) based on the normalizing flow architecture (*48*) to encode the spectra in image space *X* in a latent space *Z* in which the samples are distributed according to a multivariate standard Gaussian. Let *f*_Θ_: *X* ⊂ *ℝ^n^* → *Z* ⊂ *ℝ^n^* denote the neural network with parameters Θ. Then, we can use the change of variable formula to compute the log likelihood log*P*(*x*∣Θ) for a spectrum *x* as

$$\log P(x|\text{Θ})=-\frac{1}{2}∥f_{\text{Θ}}(x){∥}_{2}^{2}-\frac{n}{2}\log(2\pi )+\log|\det Jf_{\text{Θ}}(x)|$$ (2)

where *Jf*_Θ_ denotes the network’s Jacobian, and we already assume that the latent space *Z* is normally distributed.

The change of variable formula enables us to compute log*P*(*x*∣Θ) for a single neural network. To be able to compute the WAIC variance and expectation term, we generate an ensemble of networks (default: *n* = 5) with identical architecture but different random seeds.

#### Ischemia index

As not only the inference but also the training needs to be performed during the actual surgical procedure, we faced the requirement of fast training times (seconds rather than minutes or hours). To achieve fast network convergence based on the patient-individual data, we pretrained our neural network ensemble on simulated data. Specifically, we simulated light transport in tissue using an MC method to compute high-resolution spectra covering a large space of physiological states (see the “Implementation details” section). In the second step, these high-resolution spectra were adapted to the MSI sensor taking the light source, filter responses, and other transmission spectra into account. The fully trained network then allows us to compute WAIC(*x*) for any new spectrum *x*. Higher WAIC values imply that the spectrum is OoD, i.e., ischemic. As the recorded organ of interest consists of more than one multispectral pixel, we postprocess the WAIC values by aggregating them over ROIs. This leads to a single value per MSI frame, which we refer to as the ischemia index.

Note that we presented the general idea of using INNs for OoD in the context of uncertainty quantification at a conference workshop (*49*). However, the idea of phrasing the ischemia detection problem as an OoD problem is entirely novel (patent pending). The concrete instantiation of this approach for ischemia detection in partial nephrectomy is provided in the “Implementation details” section, including all relevant implementation details.

### Patient study

The aim of this study was to investigate the feasibility of our approach to contrast agent–free real-time ischemia monitoring. In this section, we describe our dataset (see the “Patient data” section), the instantiation of our ischemia detection method (see the “Automatic ischemia detection” section), and the visualization and evaluation techniques (see the “Assessment methods” section).

#### Patient data

Here, we describe the patient recruitment, image acquisition process, technical inclusion criteria for our evaluation, and our data splits.

##### Patient recruitment

All patients were recruited in the Städtisches Klinikum Karlsruhe (Karlsruhe, Germany). The experiments involving humans were performed in accordance with the Declaration of Helsinki, and all protocols were approved by the Landesärztekammer Baden-Württemberg (DE/EKBW01, study reference number: B-F-2019-101). The inclusion criteria for all subjects were as follows: (i) Subjects were undergoing partial nephrectomy, and (ii) subjects were adults (≥18 years old). This resulted in a total of 10 subjects with an age range between 40 and 82 years old, 7 of which were male and 3 were female. Table 2 shows an overview of all the subjects recruited for this study.

**Table 2.** Patients recruited in our clinical study. ccRCC, clear cell renal cell carcinoma; PRCC, papillary renal cell carcinoma; ChRCC, chromophobe renal cell carcinoma; BMI, body mass index.

ID	Age	BMI	Sex	Tumor type	Smoker	Diabetic	Comorbidities	Data split
1	80	25	Male	PRCC	No	No	Prostate cancer	Retrospective
2	70	22	Female	Benign oncocytoma	No	No	Psychiatric illness	Retrospective
3	49	40	Male	ccRCC	No	No		Retrospective
4	56	30	Male	Benign oncocytoma	No	No		Retrospective
5	49	49	Female	Leiomyoma	No	Yes	Depression	Retrospective
6	82	22	Male	ccRCC	No	No	Sigma carcinoma	Prospective
7	67	29	Male	ccRCC	Yes	No		Prospective
8	56	43	Male	PRCC	No	No		Prospective
9	53	31	Male	ChRCC	No	No	Rheumatism	Prospective
10	40	24	Female	Angiomyolypoma	No	No	Migraine	Prospective

##### In vivo image acquisition

Recordings were taken from subjects undergoing partial nephrectomy at the Städtisches Klinikum Karlsruhe. The standard procedure involves the generation of six surgical ports, three of which were dedicated to the da Vinci robotic arms (Intuitive Surgical Inc., California, USA), one of which was used for the da Vinci RGB surgical camera, and the remaining two as assisting ports used for other instruments (e.g., scissors, clip appliers, etc.).

**Fig. 8.**
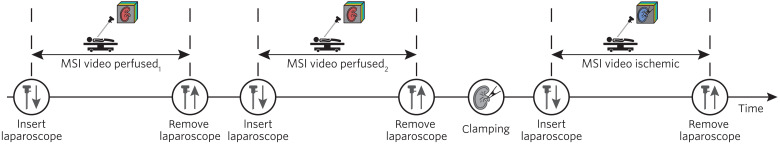
MSI recording procedure in the OR. We recorded two MSI sequences [perfused_1_ (for training) and perfused_2_ (for testing)] before the clamp was applied to the renal artery and one sequence after applying the clamp (for testing). The laparoscope was removed and reinserted in the patient’s abdominal cavity before recording the testing sequence for perfused kidney to obtain the same conditions as for the ischemic kidney.

The conventional workflow was adapted as follows for our measurements. After removing the fatty tissue from the surface of the kidney and locating the renal artery, the multispectral laparoscope was inserted through one of the assisting ports and three measurements were performed to enable training and validation of our ischemia detection algorithm. Each measurement was performed for 45 s, yielding ≈1200 recorded MSI images (Fig. 8):

1) First recording of perfused kidney (training sequence: perfused_1_): MSI images of the surface of the kidney were recorded before a clamp was applied to the renal artery. Part of these data was used for training the personalized ischemia index, as detailed in the “Implementation details” section.

2) Second recording of perfused kidney (testing sequence: perfused_2_): To simulate unsuccessful clamping, an additional image sequence was recorded before the clamp was applied. For this purpose, the multispectral laparoscope was removed from the surgical port and reinserted through the same port. Then, MSI images of the surface of the kidney were again recorded. The reinsertion of the laparoscope was performed to obtain slightly different acquisition conditions. This is also the case when actual clamping is performed, as the laparoscope needs to be removed before the renal artery can be clamped through the same surgical port (see the next paragraph).

3) Recording of ischemic kidney (testing sequence: ischemic): The MSI laparoscope was removed from the abdominal cavity, such that the surgical team could perform the clamping procedure through the same port used for the laparoscope. After clamping of the renal artery, the MSI laparoscope was reinserted in the abdominal cavity, and the surgical team attempted to acquire MSI data from a kidney region as close as possible to that before the clamping. Last, ICG was injected, and the resulting fluorescence signal from the Firefly system of the da Vinci robot served as the basis for determining whether the clamping had been successful. After the surgery, the Firefly recordings were used to verify that the recorded region of the kidney was ischemic. Note that this is straightforward because in all our patients, the entire visible surface of the kidney was ischemic, as confirmed by a lack of ICG signal. The ICG injection was prepared by mixing 50 mg of ICG powder (PULSION Medical Systems SE, Feldkirchen, Germany) with 10 ml of distilled water.

To obtain high-quality data, all measurements were performed on parts of the tissue in which regions without blood and fatty tissue could be observed. Before each measurement, (i) the camera integration time was set to 40 ms, (ii) the laparoscope was positioned to include the clean surface of the kidney in the field of view of the camera, and (iii) the intensity of the light source was adjusted to minimize the number of specular reflections observed on the surface of the kidney. Furthermore, the light source of the da Vinci RGB surgical camera was turned off during all our measurements.

##### Study cohort

To obtain a reliable reference for our measurements, we only included patients in which the ischemia induction of the kidney was successfully confirmed by ICG injection as part of the traditional surgical procedure. Furthermore, we omitted patients for which image recordings were underexposed because of a faulty light guide. A common flaw in machine learning–based image analysis is the overfitting on the test data (*50*). Even if an algorithm is strictly trained on the training data, a common procedure involves testing multiple different models or hyperparameter configurations on the test set and then reporting the best model. To avoid an overestimation of algorithm performance resulting from such practice, we finalized the complete algorithm based on existing datasets before conducting the patient study. The only exception was the number of MSI frames used for training the networks used to compute the ischemia index. To fix this hyperparameter, we separated the recruited patients into a retrospective and prospective split. The retrospective data split was composed of the first half of the patients (5) and the prospective split of the second half of the patients (5). The number of MSI frames was determined using only the retrospective dataset.

#### Automatic ischemia detection

To instantiate our ischemia monitoring approach (see the “Algorithm for live ischemia monitoring” section) for partial nephrectomy, the following design decisions needed to be made: (i) How to preprocess the raw camera data before providing it to the neural network ensemble and (ii) how to aggregate values over individual spectra to derive an image-level ischemia index at test time.

##### Data preprocessing

We decided to represent the tissue state in an MSI image by two ROIs that correspond to regions on the tissue from which high-quality measurements can be obtained. The ROIs were chosen by the physician or technical assistant at the beginning of a recording sequence according to the process detailed in the “Implementation details” section. As we implemented an automatic ROI tracking algorithm, the ROIs only had to be chosen once at the beginning of each sequence. The data from each ROI was first normalized with a white (*W*) and dark (*D*) reference recording taken with a highly reflective target (Spectralon, Edmund Optics, Barrington, USA). Given an ROI of dimensions *N* × *M* × *B*, where (*N*, *M*) are the spatial dimensions and *B* is the number of spectral bands, the intensity *I* of each pixel at spatial location (*i*, *j*) and spectral band *k* was normalized according to

$${\left[{\bar{I}}_{\left(i,j\right)}\right]}_{k}=\frac{{\left[I_{\left(i,j\right)}\right]}_{k}-{\left[D_{\left(i,j\right)}\right]}_{k}}{{\left[W_{\left(i,j\right)}\right]}_{k}-{\left[D_{\left(i,j\right)}\right]}_{k}}$$ (3)

Subsequently, an ℓ_2_ normalization across different bands was performed to compensate for the influence of light source intensity changes due to changes in the distance of the laparoscope to the surface of the kidney. The resulting spectra ${\left[{\hat{I}}_{\left(i,j\right)}\right]}_{k}$ can be compared between different image sequences. The normalized spectra $\hat{I}$ were then used to train our ensemble of INNs, and the median normalized spectra within each ROI were used for the rest of our analysis.

##### Ischemia index

Our ischemia monitoring approach leveraged spectral information from two different ROIs. To compute our ischemia index from this information, we first aggregated WAIC values belonging to the same ROI

WAIC(ROI)medianWAIC[x(i,j)]i=1,…,Nj=1,…,M (4)

where (*i*, *j*) is taken over the spatial dimensions of the ROI of shape *N* × *M*.

Then, we aggregated the two ROIs per frame via the mean to obtain the final ischemia index

$$\text{Ischemia index}\;(\mathrm{Frame})\frac{1}{2}\{\mathrm{WAIC}[\mathrm{RO}I_{1}(\mathrm{Frame})]+\mathrm{WAIC}[{\mathrm{ROI}}_{2}(\mathrm{Frame})]\}$$ (5)

For ease of visualization, we min-max–normalized the ischemia index for each patient individually. As this is a strictly monotonic transformation, this has no influence on the AU-ROC metric.

#### Assessment methods

This section introduces our methods for assessing tissue heterogeneity and for validating our ischemia index.

##### Statistical analysis of tissue heterogeneity

For two-dimensional visualization of the high-dimensional spectral data, the data from each ROI was first normalized according to the procedure described in the “Automatic ischemia detection” section, yielding a median spectrum per ROI, frame, state, and subject. For each frame, we averaged the median spectra across all ROIs, computed the first two PCs based on the data from all subjects and projected the data points onto these new axes (*32*).

We further computed the proportion of explained variability in reflectance by different components using linear mixed models, as suggested by Schreck and Wiesenfarth (*51*). Given a patient index *i* = 1, …,10, and an MSI frame index *j* = 1, …, *n_i_*, we fitted the following model for each wavelength separately

$$r_{ij}=\alpha +\beta S_{ij}+\delta_{i}+\epsilon_{ij}$$ (6)

where *r* represents the averaged median reflectance across tracked ROIs for a given wavelength (see the “Implementation details” section). *S_ij_* is an indicator variable, indicating the perfusion state of the kidney (1 for ischemic or 0 for perfused). Furthermore, α represents an intercept for each linear model, β is a fixed state effect, δ_*i*_ ~ $N(0,\sigma_{\delta }^{2})$ is a random patient effect, and $\epsilon_{ij}$ ~ $N(0,\sigma_{\epsilon }^{2})$ are residuals, with respective variances $\sigma_{\delta }^{2}$ and $\sigma_{\epsilon }^{2}$ that are assumed to be independently normally distributed. We computed the proportions of explained variability following (*51*).

#### Validation of the ischemia index

To validate the ischemia index, we designed the recording process such that we had comparable testing sequences for perfused and ischemic kidney as detailed in the “Patient data” section. For performance assessment, we computed the ischemia index for the first 70 frames of both testing sequences. Using ischemic as the positive class, perfused as the negative class, and the ischemia index as prediction score (higher implying more ischemic), we then computed the AU-ROC as primary performance metric.

##### Analysis of example patients

For the detailed analysis of the representative patient (patient 3) and the patient on which our method failed (patient 7; Fig. 7), we computed the box plots on the same data as our network was trained and tested on; that is, all valid pixels from three kidney states (perfused_1_, perfused_2_, and ischemic), both ROIs, and the first 70 frames were used. The same data were also used for the density plots where we computed the first two PCs and then applied KDE estimation (*52*, *53*) on the projected values for each state separately via the fast KDE method (*54*, *55*).

### Implementation details

This section presents implementation details for some of the image analysis methods presented in the manuscript.

#### Invertible neural networks

The training data consisted of two ROIs of size 30 × 30 pixels located on the perfused kidney. These ROIs were tracked for 70 consecutive frames. The INNs were implemented using the PyTorch framework and the FrEIA package for invertible architectures. Following up on our previous work with INNs (*47*, *49*, *56*), we applied the normalizing flow architecture originally introduced in Dinh *et al.* (*48*) and used the following network default settings: 20 affine coupling blocks (*48*) with three-layer fully connected subnetworks with 256 hidden dimensions, rectified linear unit activations, and fixed channel permutations. The networks were trained using maximum-likelihood training, i.e., by minimizing the loss *L*(*x*) = − log *P*(*x*∣Θ) as given in Eq. 4 using the Adam optimizer, a learning rate of 10 × 10^−4^, and a weight decay of 1 × 10^−4^. The training data were *z*-score–normalized, and we used noise augmentation using additive Gaussian noise with an SD of 0.05. The pretraining based on MC simulations was performed offline with 100 epochs. Fine-tuning on the patient data was done for 10 epochs. All hyperparameters, except for the number of training frames, were optimized with preexisting data disjoint from the current study data.

#### ROI tracking

At least two ROIs of size 30 × 30 pixels were selected on each image sequence, each based on the requirements that (i) there was no adipose tissue on top of the kidney tissue, (ii) there was no blood leakage staining the kidney tissue, (iii) the camera counts were not oversaturated and not undersaturated, (iv) there was no visible smoke, and (v) the ROI locations did not overlap. Each ROI was defined in the first image and subsequently tracked across consecutive frames with a DL-based algorithm. As the multispectral laparoscope needed to be retracted between the two recordings (before and during clamping), we were not able to ensure imaging of the exact same region. Hence, the ROIs of the different sequences do not correspond.

The tracking was performed on RGB images reconstructed from the MSI images, as detailed in the “Implementation details” section. To enable reliable tracking, the RGB images were first transformed into HSV (hue, saturation, value) color space, then the V channel was normalized with contrast limited adaptive histogram equalization (CLAHE), and, finally, the resulting image was converted back to RGB. The kernel size used by CLAHE was set to one-eighth of the image height by one-eighth of its width, the number of bins was set to 256, and the clipping limit was set to 0.01.

The reconstructed and normalized RGB images were fed into a pretrained Visual Geometry Group (VGG-19) (*57*) neural network, and deep features were extracted from its seventh convolutional layer. The extracted features were further processed by the tracker discriminative correlation filter with channel and spatial reliability (CSR-DCF) (*58*). This tracker compares the features extracted from two consecutive frames to localize the ROI in the new frame.

Before using a processed video sequence (70 frames corresponding to 2 to 3 s) for training or testing, the sequence of automatically tracked ROIs was manually verified to ensure that (i) the ROIs did not disappear from the camera field of view, (ii) at least 95% of all tracked pixels were not oversaturated and not undersaturated, and (iii) there was no visible drift of the ROI from the original annotated tissue region. Those two ROIs fulfilling the criteria that could be tracked the longest were kept for further processing.

#### MC simulations

Generation of simulated tissue spectra for pretraining the ischemia index was inspired by previous work (*12*). Light transport of several tissue structures was simulated with a model composed of three infinitely wide slabs. Each slab was defined by optical and physiological parameter values of blood volume fraction *v*_hb_, blood oxygen saturation *s*, reduced scattering coefficient at 500 nm *a*_mie_, scattering power *b*_mie_, anisotropy *g*, refractive index *n*, and layer thickness *d*. Such parameters were computed on the basis of reference values from literature (*59*) and later used to create MC simulations. The reference values used from literature include extinction coefficients of deoxyhemoglobin ϵ_Hb_ and oxyhemoglobin ϵ_HbO2_, as well as absorption μ*_a_* and scattering μ*_s_* coefficients. A graphics processing unit (GPU) accelerated version (*60*) of the popular Monte Carlo multilayered (MCML) simulation framework (*61*) was chosen to generate spectral reflectances. In total, 5.5 × 10^5^ samples were simulated by simulating 10^6^ photons per wavelength in the range [λ_min_ − λ_max_]: 300 to 1000 nm, at a step size of 2 nm. The properties of each tissue layer were sampled uniformly from the range 0 to 30% for blood volume fraction *v*_Hb_, 0 to 100% for oxygenation *s*, 5 to 50 cm^−1^ for the reduced scattering coefficient *a*_mie_, 0.3 to 3 arbitrary units (a.u.) for the scattering power *b*_mie_, 0.80 to 0.95 a.u. for anisotropy *g*, 1.33 to 1.54 a.u. for the refractive index *n*, and 0.002 to 0.2 cm for the tissue layer thickness *d*. On the basis of such properties, the absorption and scattering of each sample were computed based on Eqs. 7 and 8

$$\mu_{a}(v_{\mathrm{Hb}},s,\lambda )=\frac{v_{\mathrm{Hb}}[s\cdot \epsilon_{\mathrm{Hb}O2}(\lambda )+(1-s)\cdot \epsilon_{\mathrm{Hb}}(\lambda )]\cdot \ln(10)\cdot 150g\;{\mathrm{liter}}^{-1}}{6.45\cdot 10^{4}{\text{g mol}}^{-1}}$$ (7)

$$\mu_{s}(a_{\mathrm{mie}},b,\lambda )=\frac{a_{\mathrm{mie}}}{1-g}{\left(\frac{\lambda }{500\;\mathrm{nm}}\right)}^{-b_{\mathrm{mie}}}$$ (8)

The simulated reflectances *r*(λ) were then transformed into the MSI camera measurement *r_k_* at band *k* according to

$$r_{k}=\frac{\int_{\lambda_{\min}}^{\lambda_{\max}}T(\lambda )\cdot I(\lambda )\cdot f_{k}(\lambda )\cdot r(\lambda )d\lambda }{\int_{\lambda_{\min}}^{\lambda_{\max}}T(\lambda )\cdot I(\lambda )\cdot f_{k}(\lambda )d\lambda }$$ (9)

Here, T(λ) represents the optical transmission profile of the optical components of our hardware setup (Fig. 6), *I*(λ) is the relative irradiance of the light source, and *f_k_*(λ) characterizes the *k*th optical filter response of the camera. The transformed simulated spectra *r_k_* were used to pretrain our DL models as described in the “Automatic ischemia detection” section.

#### RGB image reconstruction

Reconstruction of RGB images from MSI was required for (i) automatic ROI tracking and (ii) illustration purposes. The reconstruction was achieved by computing a transformation matrix *T* of dimensions (3 × *B*), where *B* = 16 represents the number of bands in one MSI image. A linear regressor was fitted to compute the transformation matrix *T* based on the filter response of the MSI camera, the transmission of each optical component (Fig. 6), and the filter response of an artificial RGB camera. The artificial RGB camera was simulated by three Gaussian filters centered at 460, 550, and 640 nm, each with an SD of 42 nm. In addition, the spectral response of the MSI camera was adjusted by multiplying the transmission T of each optical component by the filter response of the MSI camera *F*_MSI_

$$F^{\prime }=F_{\mathrm{MSI}}T$$ (10)

If we consider the filter response of the artificial camera *F*_RGB_ and the spectral response of the MSI camera *F*′ after correcting for the optical components integrated in the system, we can compute the transformation matrix as

$$\begin{array}{rl} T_{\min} & =\underset{T^{\prime }}{\text{arg min}}(∥F_{\mathrm{RGB}}-F^{\prime }T^{\prime }{∥}_{2})\\ T & =T_{\min}⊘N \end{array}$$ (11)

where *T* represents the coefficients of the linear regressor, ⊘ denotes component-wise division, and *N* is a normalization vector with three elements, which can be computed as

$$N_{i}=\sum_{j}^{B}{\left(F^{\prime }T_{\min}\right)}_{i,j}|i\in \{1,2,3\}$$ (12)

#### Optical transmission profiles

All optical transmission profiles shown in Fig. 6A were measured with a spectrometer (HR2000+, Ocean Insight, Orlando, USA). One hundred transmission measurements of each optical component were averaged and then smoothed across wavelengths by rolling window averaging with a window of ≈19 nm width. The values in the ranges 400 to 419 nm and 681 to 700 nm were ignored after smoothing to avoid unwanted border effects.

#### Beer-Lambert regression

We used the Beer-Lambert law (*37*) to estimate the total blood volume fraction (*v*_HbT_) and oxygenation (*s*) within the tracked ROIs described in the “Implementation details” section. This law states that a linear relationship exists between the absorption *a*(λ) of a medium, its attenuation coefficient μ*_a_*(λ), and the optical path length *l*

$$a(\lambda )∼-\log[r(\lambda )]=\mu_{a}(\lambda )l+g$$ (13)

where *g* is a term accounting for scattering losses. Taking into account that blood is the main absorber in internal organs, we can replace the attenuation coefficients by the extinction coefficients of blood

$$a(\lambda )=v_{\mathrm{Hb}O2}\cdot \epsilon_{\mathrm{Hb}O2}(\lambda )\cdot l+v_{\mathrm{Hb}}\cdot \epsilon_{\mathrm{Hb}}(\lambda )\cdot l+g$$ (14)

where *v* are concentrations and ϵ are the corresponding extinction coefficients. Given measurements at multiple wavelengths, Eq. 16 describes a system of linear equations, which can be solved for *v*_HbO2_ · *l* and *v*_Hb_ · *l* using ordinary least squares regression. This enables us to estimate the oxygenation *s* and the total hemoglobin concentration *v*_HbT_ via

$$s=\frac{v_{\mathrm{HbO}2}\cdot l}{v_{\mathrm{Hb}}\cdot l+v_{\mathrm{HbO}2}\cdot l}$$ (15)

$$v_{\mathrm{HbT}}\cdot l=v_{\mathrm{HbO}2}\cdot l+v_{\mathrm{Hb}}\cdot l$$ (16)

Note that estimations of *v*_HbT_ can only be computed up to a constant factor *l*. Because all extinction coefficients are given at a high spectral resolution but we use a multispectral sensor with a relatively low number of bands, we adapted the high-resolution extinction coefficients by averaging them over the filter response function of each band.

## Notes

View/request a protocol for this paper from *Bio-protocol*.
