# Supplementary material for: Spectral imaging enables contrast agent–free real-time ischemia monitoring in laparoscopic surgery
Source: Sci Adv. 2023 Mar 10;9(10):eadd6778. doi: 10.1126/sciadv.add6778 (PMC10005169; doi:10.1126/sciadv.add6778)
Supplement: Supplementary file 1 — Figs. S1 to S4 Legend for movie S1 [file sciadv.add6778_sm.pdf]

Science Advances
AAAS

Supplementary Materials for

Spectral imaging enables contrast agent–free real-time ischemia monitoring in laparoscopic surgery

Leonardo Ayala *et al.*

Corresponding author: Leonardo Ayala, l.menjivar@dkfz-heidelberg.de; Tim J. Adler, t.adler@dkfz-heidelberg.de; Lena Maier-Hein, l.maier-hein@dkfz-heidelberg.de

Sci. Adv. **9**, eadd6778 (2023)
DOI: 10.1126/sciadv.add6778

The PDF file includes:

Figs. S1 to S4
Legend for movie S1

Other Supplementary Material for this manuscript includes the following:

Movie S1

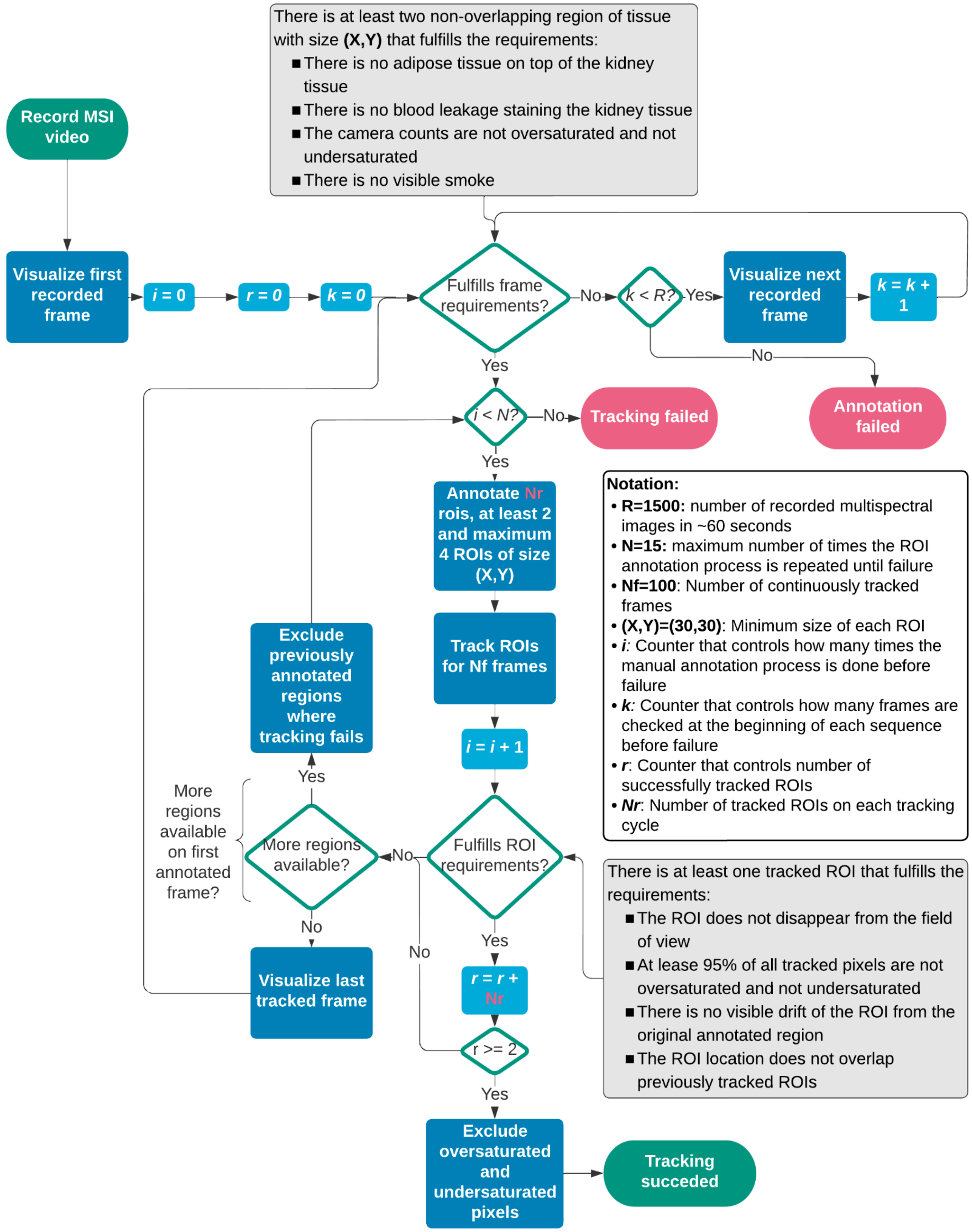

Figure S1: We annotate Regions of Interest (ROIs) in the surface of tissue and ensure high data quality. ROI annotation flowchart based on a sequence of spectral images.

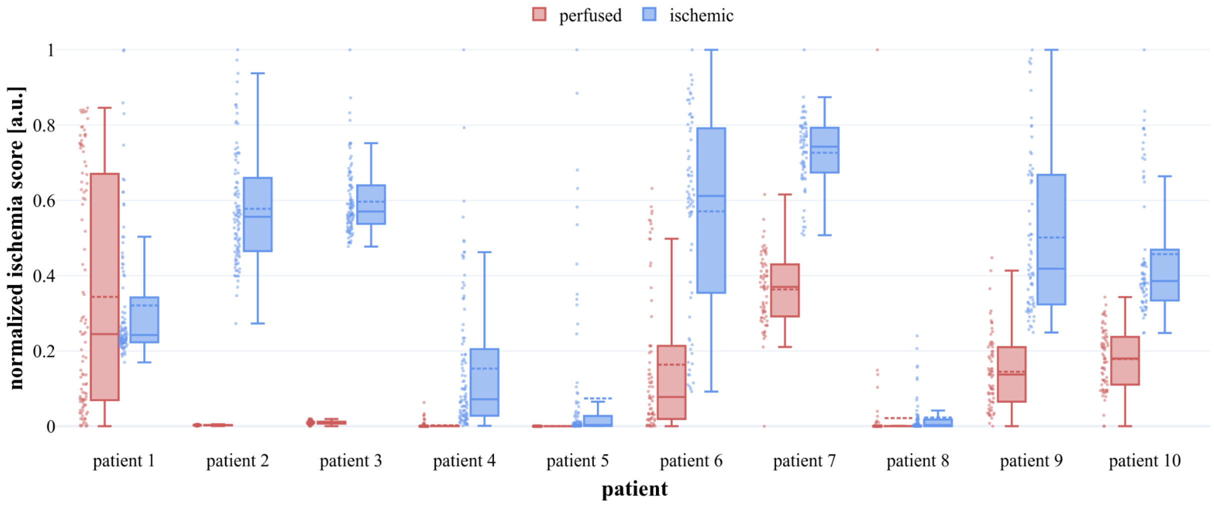

Figure S2: RGB data is not suitable for discriminating different tissue states. We reconstructed RGB data from our MSI data and calculated the *ischemia index* for every frame in video sequences of perfused and ischemic kidney separately for each patient in analogy to Figure 5.

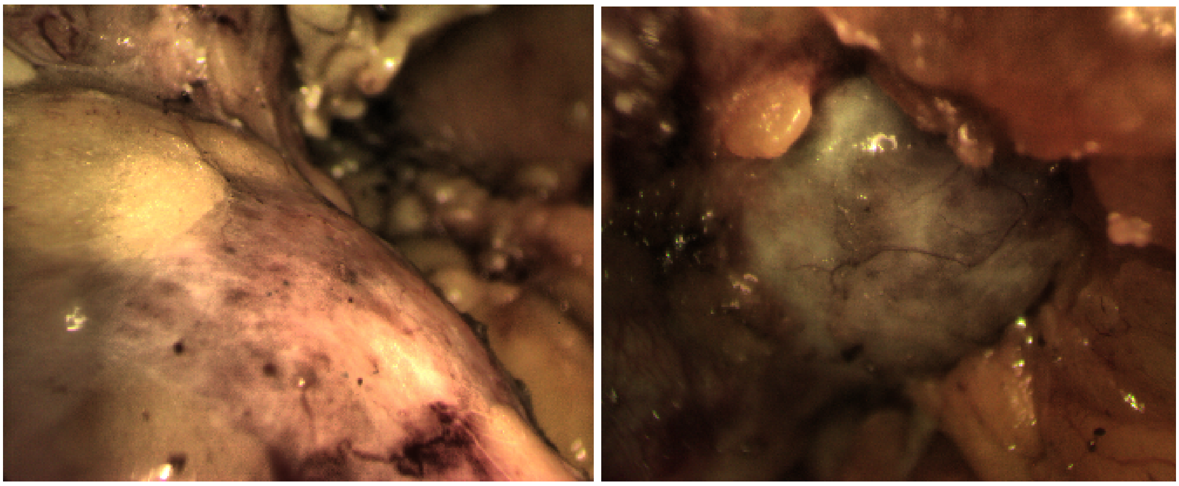

Figure S3: Reconstructed RGB images from MSI data demonstrate that our spectral system achieves high spatial resolution, thus allowing the visualization of relevant structures such as vessels. Exemplary reconstructed RGB images from patients 1 (left) and 6 (right).

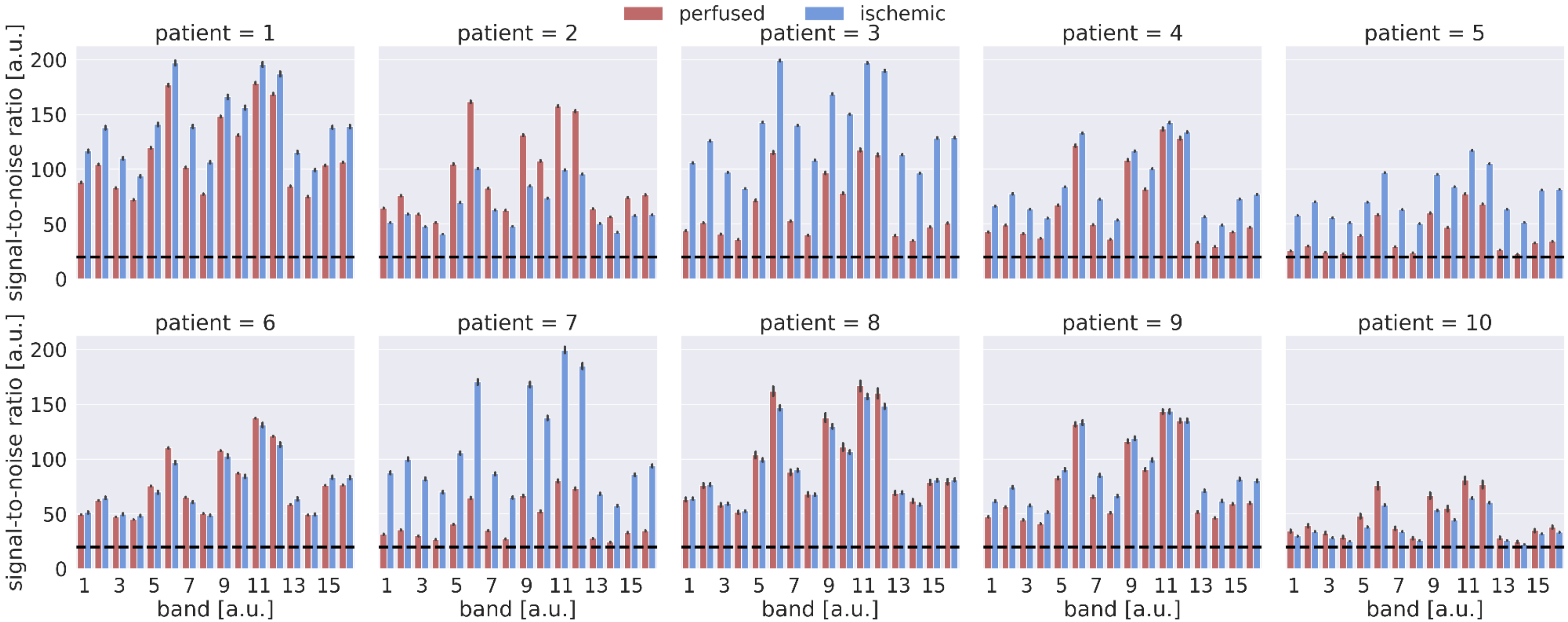

Figure S4: We ensured that the signal-to-noise ratio (SNR) of each measurement was at least 20 to obtain enough photon counts with the multispectral sensor. The horizontal dashed line corresponds to an SNR of 20. The measurements featured a median/mean SNR of 72/81.

Video S1: The ischemia index is capable of monitoring ischemia in real time from surgical videos. Exemplary animations depicting perfused (left) and ischemic (right) tissue for patient 3. Low ischemia index indicates perfused tissue, while high ischemia index represents ischemic tissue.
